# Safety and efficacy of non-reduced use of caspofungin in patients with Child–Pugh B or C cirrhosis: a real-world study

**DOI:** 10.1007/s15010-023-02162-0

**Published:** 2024-01-24

**Authors:** Shi-Dan Yuan, Ke-Li Wen, Yun-Xing Cao, Wen-Qi Huang, An Zhang

**Affiliations:** Department of Critical Care Medicine, The Second Affiliated Hospital of Chongqing Medical University, 76# Linjiang Road, Yuzhong District, Chongqing, 400016 China

**Keywords:** Caspofungin, Cirrhosis, Efficacy, Fungal infection, Safety

## Abstract

**Background and purpose:**

The need for dose adjustment of caspofungin in patients with hepatic impairment is controversial, especially for those with Child–Pugh B or C cirrhosis. The purpose of this study was to investigate the safety and efficacy of standard-dose caspofungin administration in Child–Pugh B and C cirrhotic patients in a real-world clinical setting.

**Patients and methods:**

The electronic medical records of 258 cirrhotic patients, including 67 Child–Pugh B patients and 191 Child–Pugh C patients, who were treated with standard-dose of caspofungin at the Second Affiliated Hospital of Chongqing Medical University, China, from March 2018 to June 2023 were reviewed retrospectively. The white blood cells (WBC), hepatic, renal and coagulation function results before administration and post administration on days 7, 14 and 21 were collected, and the efficacy was assessed in all patients at the end of caspofungin therapy.

**Results:**

Favorable responses were achieved in 137 (53.1%) patients while 34 (13.2%) patients died. We observed that some patients experienced an increase of prothrombin time (PT) or international normalized ratio (INR), or a decrease of WBC, but no exacerbation of hepatic or renal dysfunction were identified and no patient required dose interruption or adjustment because of an adverse drug reaction during treatment with caspofungin.

**Conclusions:**

Standard-dose of caspofungin can be safely and effectively used in patients with Child–Pugh B or C cirrhosis, and we appealed to re-assess the most suitable dosing regimen in this population to avoid a potential subtherapeutic exposure.

**Supplementary Information:**

The online version contains supplementary material available at 10.1007/s15010-023-02162-0.

## Introduction

The prevalence of liver cirrhosis has been increasing over past decades and imposes a considerable economic burden on many nations [1]. It has been well-documented that cirrhotic patients are more susceptible to opportunistic infections, including invasive fungal infection (IFI), compared to the general population and early initiation of appropriate and effective antibiotic therapy is critical for prognosis [2]. Several drugs are currently available for IFI, including amphotericin B formulations, triazoles, and echinocandins. However, owing to their outstanding tolerability/safety profile and a lower chance of inducing drug-related adverse events, echinocandins are considered as the preferred therapeutic option for IFI [3]. Caspofungin is one of the three novel class of echinocandins and undergoes liver-dependent metabolism, yet still retains the potential for organ toxicities, such as hepatotoxicity, nephrotoxicity, coagulopathy and leukopenia. Furthermore, compared to healthy persons, patients with pre-existing liver disease have an elevated susceptibility to drug-induced liver injury [4]. The product information of caspofungin recommends a dose reduction to 35 mg daily following the 70 mg loading dose in patients with Child–Pugh B [5]. There is insufficient clinical experience to develop evidence-based guideline recommendations in patients with Child–Pugh C.

In recent years, several studies have successively demonstrated that reducing the dose of caspofungin in patients with moderate to severe liver impairment may lead to suboptimal drug exposure and undesirable clinical outcomes [6–9]. However, these studies were mostly conducted in critically ill patients with concurrent acute liver dysfunction and had limited sample sizes or were case reports. The tolerability of caspofungin for patients with pre-existing liver disease, especially in cirrhosis and Child–Pugh C patients has not been well-examined previously. Therefore, the objective of this study was to investigate the safety and efficacy of using standard caspofungin dose for IFI in patients with Child–Pugh B or C cirrhosis.

## Methods

### Study population

This retrospective cohort study was conducted at the Second Affiliated Hospital of Chongqing Medical University in China from March 2018 to June 2023. Inclusion criteria included met the criteria for the diagnosis of liver cirrhosis, suspected or confirmed fungal infection, received a maintenance dose of 50 mg daily caspofungin therapy. Exclusion criteria included age < 18 years, absence of cirrhosis, Child–Pugh score < 7 points or cannot be calculated, caspofungin treatment duration < 7 days, treated with the reduced dosage of caspofungin, or received a liver transplant during hospital stay.

### Data collection

Patients’ laboratory results including the total protein (TP), albumin, alanine aminotransferase (ALT), aspartate amino transferase (AST), alkaline phosphatase (ALP), γ-gamma-glutamyl transpeptidase (γ-GGT), total bilirubin (TBIL), direct bilirubin (DBIL), prothrombin time (PT), activated partial thromboplastin time (APTT), international normalized ratio (INR), serum creatinine (Scr), glomerular filtration rate (GFR) and white blood cells (WBC) were collected before initiation of treatment (D0) and post treatment on the 7th day (D7), the 14th day (D14) and the 21th day (D21). In the meantime, demographic characteristics, comorbid diseases, whether received human albumin (HA) infusion or anti-inflammatory and liver-protective agents were also collected.

### Efficacy measurements

Responses to antifungal therapy in patients with IFI was defined according to the Mycoses Study Group and European Organization for Research and Treatment of Cancer Consensus Criteria that can be characterized into treatment success which includes complete response as well as partial response, and treatment failure which includes stable response, progression of fungal disease and death [10].

### Statistical analysis

All analyses were performed using SPSS 22.0 statistical software (SPSS Inc., Chicago, IL, USA). The Kolmogorov–Smirnov test was used to determine the normality of the numeric data. Normally distributed data were expressed as means ± standard deviation, and analyzed by the Student’s *t* test, otherwise they were represented as the median (interquartile range), and analyzed by the Mann–Whitney *U* test. Categorical variables were displayed as frequencies (percentages), and the Chi-square test was used to analyze relationships between groups. Friedman test was utilized to compare the changes in various parameters at three or more different timepoints, and then Wilcoxon signed-rank test was applied for comparisons between the two timepoints. For all analyses, *P* ≤ 0.05 was considered statistically significant.

## Results

### Patient characteristics

**Fig. 1:**
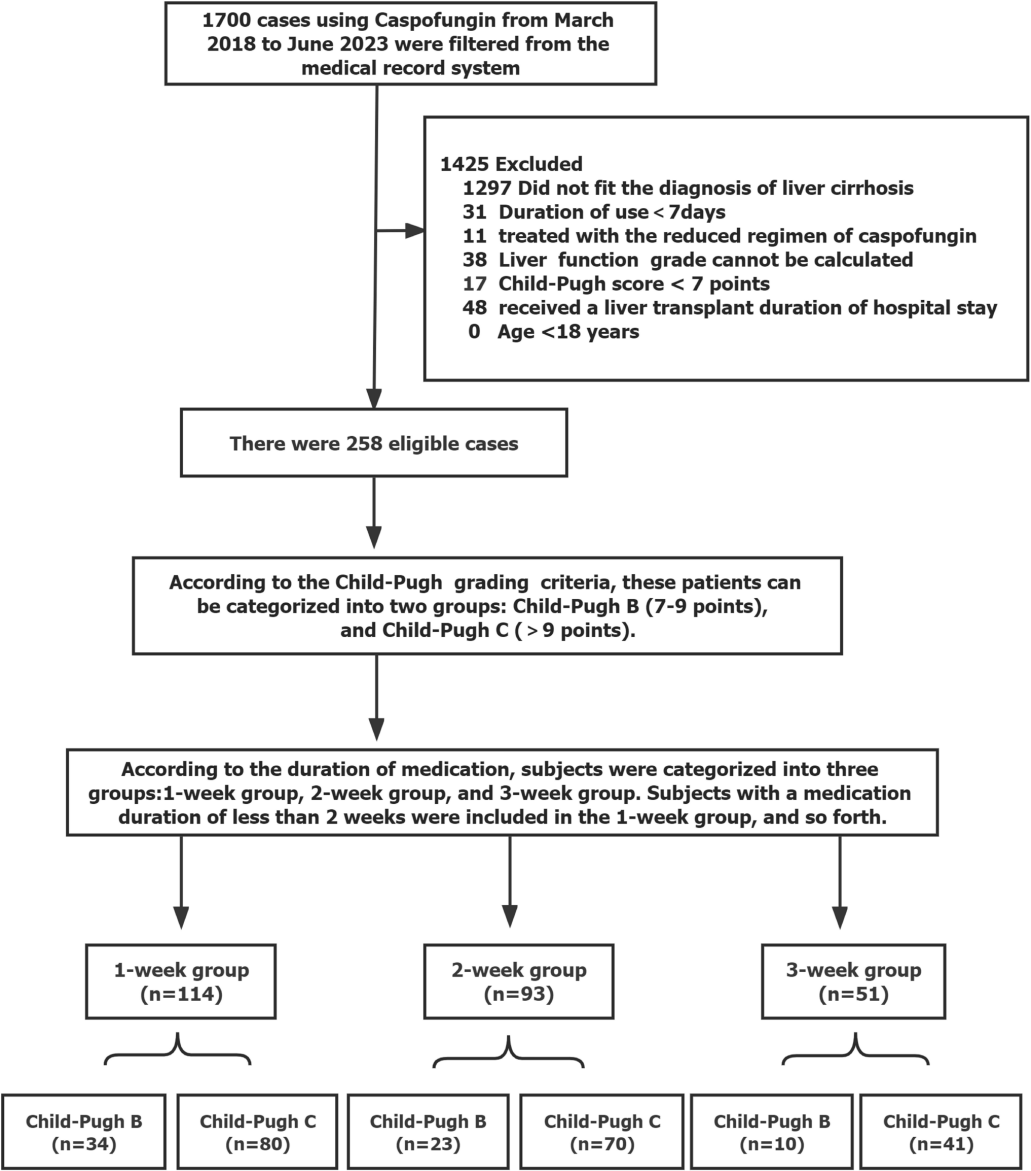
Flow chart of patients recruitment

A total of 1700 patients received caspofungin therapy were screened and 258 patients were eventually included in the study, including 67 Child–Pugh B cirrhosis patients and 191 Child–Pugh C cirrhosis patients. Further details are provided in Fig. 1.

Patients’ baseline and demographic characteristics were presented in Online Resource 1, Electronical supplement material (ESM) 1. The mean age of the patients was 57.5 ± 12.0 years and median weight were 60.0 kg (51.0, 66.1), and the proportion of male patients (72.5%) was greater than that of female patients (27.5%). The mean albumin and mean TBIL were 30.3 g/L (28.1, 32.9) and 132.4 umol/L (46.1, 310.9), respectively. Compared to Child–Pugh B cirrhotic patients, Child–Pugh C cirrhotic patients had lower albumin (*P* = 0.001) and higher TBIL (*P* < 0.001) levels.

Common comorbidities were tumour (20.5%), diabetes (18.2%), hypertension (17.8%), haematological malignancy (15.1%), and coronary heart disease (10.9%). The most frequent primary site of infection was intra-abdominal (38.4%), followed by the pulmonary (31.8%) and oral cavity (13.6%). Furthermore, most patients were treated at the department of infectious diseases (87.6%).

In this study, viral infection (61.6%) was the leading cause of liver cirrhosis, while other common etiologies include autoimmune (12.4%), alcoholic (10.1%), cholestatic (7.4%). There were 114 confirmed IFI cases, 105 clinically diagnosed cases, and 39 suspected fungal infection cases, respectively. Patients received caspofungin for greater than or equal to 1 week but less than 2 weeks were assigned to the 1-week group, and so forth. Ultimately, there were 114 patients in the 1-week group, 93 patients in the 2-week group, and 51 patients in the 3-week group. Most of patients received anti-inflammatory and liver-protective agents and/or human albumin infusion during the period of caspofungin treatment in this study.

### Treatments

The mean treatment duration was 14.9 days (range 7–70 days) for the 258 patients. An initial 70 mg loading dose followed by a 50 mg/day maintenance dose was administered in 252 cases. Five patients received an initial 100 mg loading dose followed by 50 mg/day maintenance regimen. One patient was maintained solely on 50 mg/day caspofungin.

### Safety evaluations

A total of 30 (11.6%) patients had hepatic encephalopathy (HE) at the initiation of caspofungin, with complete reversal achieved in ten patients during treatment. Nevertheless, four patients experienced exacerbations or deterioration of HE, for which precipitating events were acute gastrointestinal hemorrhage, abdominal paracentesis, or fulminant sepsis, rather than caspofungin use.

Our results showed that the ALT level in Child–Pugh B and C patients and the γ-GGT level in Child–Pugh C patients were significantly decreased (*P* < 0.05), while the PT and INR levels in Child–Pugh B and C patients were significantly elevated on D7 (*P* < 0.05) among patients in the 1-week group (Table 1).

**Table 1:** Comparison of changes in hepatic, renal, and coagulation function among patients with different Child–Pugh scores in the 1-week group

Group	Time	TP g/L	Albumin g/L	ALT U/L	AST U/L	ALP U/L	γ-GGT U/L	TBIL umol/L	DBIL umol/L	PT S	APTT S	INR	Scr umol/L	GFR mL/min	WBC 10^9^/L
B (*n* = 34)	D0	61.8 (56.6, 68.5)	32.3 (29.0, 35.9)	38.5 (13.8, 68.3)	48.5 (32.0, 98.0)	113.0 (76.8, 165.8)	84.0 (47.0, 172.8)	25.6 (17.1, 47.2)	15.2 (8.7, 30.9)	15.6 (14.8, 18.4)	45.1 (39.8, 49.6)	1.2 (1.1, 1.5)	62.0 (50.4, 88.7)	103.2 (71.9, 131.4)	5.7 (4.3, 9.0)
	D7	62.7 (57.2, 70.7)	31.0 (29.2, 35.3)	20.0 (9.5, 58.5)	41.0 (26.0, 84.0)	110.0 (84.0, 134.0)	83.0 (51.5, 114.0)	24.5 (12.5, 57.8)	13.8 (6.9, 35.5)	17.8 (15.4, 19.3)	46.8 (42.3, 50.7)	1.5 (1.2, 1.6)	68.3 (52.5, 97.5)	90.6 (46.4, 149.2)	6.4 (4.8, 10.1)
	*Z*	− 0.524	− 0.444	− 3.150	− 0.565	− 0.216	− 1.406	− 1.412	− 1.662	− 2.400	− 0.608	− 2.582	− 1.153	− 1.325	− 0.216
	*p* value	0.600	0.657	0.002	0.572	0.829	0.160	0.158	0.096	0.016	0.543	0.010	0.249	0.185	0.829
C (*n* = 80)	D0	55.0 (51.7, 61.9)	30.3 (28.5, 32.2)	32.0 (18.3, 76.5)	73.5 (37.8, 143.8)	109.0 (76.3, 156.3)	59.5 (31.0, 123.8)	197.8 (75.1, 386.3)	140.2 (54.0, 292.4)	24.0 (20.3, 28.1)	55.1 (47.1, 63.1)	2.2 (1.8, 2.6)	72.6 (57.8, 100.3)	98.0 (66.3, 123.8)	6.8 (4.5, 10.3)
	D7	54.6 (47.7, 62.0)	31.5 (28.6, 34.1)	32.0 (16.0, 68.0)	70.0 (38.0, 116.0)	104.0 (72.0, 142.0)	52.0 (28.0, 96.0)	193.2 (69.5, 377.7)	156.1 (56.9, 300.0)	24.9 (20.0, 32.4)	59.4 (50.8, 68.8)	2.3 (1.7, 3.2)	81.2 (55.4, 123.0)	81.9 (51.0, 132.8)	6.5 (4.0, 9.3)
	*Z*	− 0.649	− 1.060	− 2.579	− 1.578	− 1.767	− 2.127	− 0.354	− 0.025	− 2.569	− 1.917	− 2.587	− 1.877	− 0.988	− 0.258
	*p* value	0.516	0.289	0.010	0.115	0.077	0.033	0.723	0.980	0.010	0.055	0.010	0.061	0.323	0.796

Data are shown as median (interquartile range)

*TP* total protein; *ALT* alanine aminotransferase; *AST* aspartate amino transferase; *ALP* alkaline phosphatase; *γ-GGT* γ-gamma-glutamyl transpeptidase; *TBIL* total bilirubin; *DBIL* direct bilirubin; *PT* prothrombin time; *APTT* activated partial thromboplastin time; *INR* international normalized ratio; *Scr* serum creatinine; *GFR* glomerular filtration rate; *WBC* white blood cells

At the same time, the levels of albumin, ALT, ALP and γ-GGT in Child–Pugh C patients changed significantly with time among patients in the 2-week group (Table 2). More specifically, we found that the albumin level on D14 was significantly higher than D0, while the levels of ALT, ALP and γ-GGT on D7 and D14 were significantly decreased than D0, and the ALT level on D14 significantly decreased than D7 (*P* < 0.05).

**Table 2:** Comparison of changes in hepatic, renal, and coagulation function among patients with different Child–Pugh scores in the 2-week group

Group	Time	TP g/L	Albumin g/L	ALT U/L	AST U/L	ALP U/L	γ-GGT U/L	TBIL umol/L	DBIL umol/L	PT S	APTT S	INR	Scr umol/L	GFR mL/min	WBC 10^9^/L
B (*n* = 23)	D0	61.9 (49.8, 73.3)	31.2 (29.7, 36.4)	26.0 (18.0, 36.0)	50.0 (26.0, 74.0)	140.0 (115.0, 300.0)	126.0 (68.0, 158.0)	30.2 (16.2, 81.4)	14.2 (8.7, 26.4)	15.4 (14.3, 17.4)	39.0 (37.0, 48.6)	1.2 (1.1, 1.4)	68.3 (48.9, 124.9)	117.2 (38.3, 164.3)	7.0 (5.2, 9.0)
	D7	59.2 (53.5, 67.4)	31.0 (29.8, 34.5)	26.0 (9.0, 37.0)	46.0 (29.0, 116.0)	134.5 (105.8, 308.0)	119.0 (50.0, 194.0)	36.3 (26.6, 78.8)	19.3 (12.7, 65.0)	17.0 (14.5, 18.9)	42.9 (40.2, 50.5)	1.4 (1.1, 1.6)	66.9 (48.9, 77.5)	113.1 (50.3, 165.5)	6.8 (4.8, 11.5)
	D14	61.8 (50.8, 62.8)	34.0 (28.8, 38.4)	29.0 (11.0, 61.0)	59.0 (36.0, 155.0)	147.0 (95.5, 260.0)	108.0 (40.0, 259.0)	37.7 (22.8, 107.3)	21.0 (13.0, 76.3)	17.0 (14.8, 17.6)	41.3 (40.6, 47.6)	1.4 (1.1, 1.5)	67.0 (50.9, 79.5)	124.9 (56.8, 165.5)	6.1 (3.4, 13.4)
	*Z*	2.000	2.533	0.644	0.456	1.209	0.133	0.533	2.235	0.250	2.800	0.424	1.067	4.000	1.895
	*p* value	0.368	0.282	0.725	0.796	0.546	0.936	0.766	0.327	0.882	0.247	0.809	0.587	0.135	0.388
C (*n* = 70)	D0	55.9 (52.6, 63.1)	29.4 (27.0, 31.9)	38.0 (25.0, 78.0)	72.0 (44.5, 118.0)	115.0 (84.0, 150.0)	54.0 (33.3, 125.3)	206.4 (90.3, 351.0)	114.2 (71.0, 265.0)	23.6 (18.9, 27.9)	53.9 (48.3, 63.3)	2.1 (1.6, 2.7)	74.0 (57.5, 93.4)	89.0 (68.7, 138.3)	6.9 (4.0, 10.1)
	D7	53.0 (49.5, 60.3)	30.1 (27.8, 32.3)	31.5 (20.8, 54.0)*	62.0 (39.5, 90.5)	108.0 (84.5, 141.0)*	51.0 (31.3, 92.0)*	171.5 (67.5, 325.3)	115.1 (49.9, 227.3)	24.6 (21.1, 31.2)	54.9 (49.4, 66.5)	2.3 (1.8, 3.0)	69.3 (49.8, 95.6)	87.2 (66.6, 149.5)	6.0 (3.8, 8.9)
	D14	55.1 (52.2, 63.0)	31.3 (28.2, 33.7)*	29.0 (19.0, 44.0)*^#^	54.0 (39.0, 92.0)	99.5 (80.3, 128.3)*	46.5 (30.0, 92.3)*	150.9 (71.2, 285.9)	105.7 (48.1, 211.2)	23.7 (19.2, 33.0)	54.7 (46.3, 66.8)	2.1 (1.6, 3.2)	73.3 (51.1, 101.0)	80.3 (64.3, 148.0)	5.7 (3.9, 8.2)
	*Z*	0.649	8.350	25.779	5.396	7.822	13.617	5.687	5.448	4.318	1.303	3.354	0.555	0.151	5.221
	*p* value	0.723	0.015	< 0.001	0.067	0.020	0.001	0.058	0.066	0.115	0.521	0.187	0.758	0.927	0.073

Data are shown as median (interquartile range)

*TP* total protein; *ALT* alanine aminotransferase; *AST* aspartate amino transferase; *ALP* alkaline phosphatase; *γ-GGT* γ-gamma-glutamyl transpeptidase; *TBIL* total bilirubin; *DBIL* direct bilirubin; *PT* prothrombin time; *APTT* activated partial thromboplastin time; *INR* international normalized ratio; *Scr* serum creatinine; *GFR* glomerular filtration rate; *WBC* white blood cells

*Compared with D0, the difference was statistically significant

^#^Compared with D7, the difference was statistically significant

On the other hand, the levels of TP, albumin, ALT, AST and WBC in Child–Pugh C patients changed significantly with time among patients in the 3-week group (Table 3). In greater detail, we found that the levels of TP on D14 and D21 were significantly higher than D7, and the TP level on D21 was significantly higher than D0. The levels of albumin on D7, D14 and D21 were significantly higher than D0, while the levels of ALT, AST and WBC on D7, D14 and D21 were significantly decreased than D0, and the levels of ALT on D14 and D21 were significantly decreased than D7, respectively (*P* < 0.05).

**Table 3:** Comparison of changes in hepatic, renal, and coagulation function among patients with different Child–Pugh scores in the 3-week group

Group	Time	TP g/L	Albumin g/L	ALT U/L	AST U/L	ALP U/L	γ-GGT U/L	TBIL umol/L	DBIL umol/L	PT S	APTT S	INR	Scr umol/L	GFR mL/min	WBC 10^9^/L
B (*n* = 10)	D0	47.0 (43.1, 72.4)	31.3 (28.4, 37.1)	25.5 (11.5, 113.25)	39.0 (20.5, 76.5)	25.5 (11.5, 113.3)	66.5 (21.8, 166.3)	78.2 (34.6, 385.8)	62.6 (23.1, 322.8)	16.3 (13.9, 18.7)	45.6 (42.9, 52.7)	1.3 (1.1, 1.8)	49.6 (41.6, 107.7)	137.0 (60.7, 195.7)	4.2 (1.5, 7.7)
	D7	55.1 (39.7, 57.5)	32.2 (29.1, 35.0)	25.5 (7.8, 83.5)	47.5 (22.3, 107.0)	25.5 (7.8, 83.5)	52.5 (24.3, 220.3)	60.7 (31.1, 117.6)	52.2 (17.2, 95.5)	16.1 (14.9, 18.5)	46.4 (40.3, 53.2)	1.3 (1.2, 1.5)	53.4 (40.9, 85.0)	120.8 (87.9, 200.4)	3.8 (2.5, 4.8)
	D14	55.1 (38.0, 69.0)	32.0 (29.5, 36.8)	31.0 (8.5, 55.0)	49.0 (17.5, 90.0)	31.0 (8.5, 55.0)	52.5 (21.5, 223.3)	49.2 (31.3, 74.7)	40.1 (17.1, 63.0)	16.3 (14.2, 27.2)	41.8 (40.1, 55.5)	1.3 (1.1, 5.3)	52.2 (36.9, 89.1)	120.8 (83.2, 211.1)	3.4 (2.0, 5.1)
	D21	50.8 (35.0, 58.1)	33.0 (30.2, 35.3)	21.0 (7.3, 71.3)	47.5 (17.3, 87.5)	21.0 (7.3, 71.3)	48.5 (18.0, 334.3)	52.0 (28.4, 182.9)	45.8 (15.2, 140.0)	16.5 (14.4, 30.4)	44.5 (30.1, 47.9)	1.3 (1.1, 6.0)	62.7 (39.7, 81.7)	121.5 (81.1, 204.4)	3.4 (1.3, 5.5)
	*Z*	3.735	1.633	3.932	1.885	3.932	1.385	6.038	5.962	2.000	2.288	3.200	0.304	0.304	4.652
	*p* value	0.292	0.652	0.269	0.597	0.269	0.709	0.110	0.113	0.572	0.515	0.362	0.959	0.959	0.199
C (*n* = 41)	D0	55.8 (50.4, 64.8)	30.0 (27.8, 31.7)	51.0 (32.0, 116.0)	81.0 (51.0, 195.0)	105.0 (56.0, 159.0)	82.0 (36.5, 159.0)	239.9 (95.9, 393.4)	193.7 (49.7, 292.0)	23.5 (18.0, 28.1)	52.6 (46.3, 62.9)	2.0 (1.5, 2.7)	68.7 (53.3, 117.9)	111.2 (70.7, 148.7)	6.5 (4.9, 10.3)
	D7	58.0 (51.9, 63.6)	31.1 (28.6, 34.8)*	36.0 (17.0, 71.5)*	67.0 (40.5, 97.5)*	101.0 (57.0, 162.0)	72.5 (37.5, 120.3)	216.8 (92.5, 374.3)	192.5 (48.2, 253.4)	24.9 (18.7, 28.6)	57.6 (48.4, 69.9)	2.3 (1.5, 2.7)	68.3 (48.1, 88.8)	93.6 (76.4, 148.7)	5.2 (3.7, 6.6)*
	D14	59.3 (55.3, 69.3)^#^	31.0 (29.4, 34.7)*	28.0 (16.5, 54.5)*^#^	60.0 (44.5, 95.0)*	108.0 (61.5, 147.5)	68.0 (39.0, 108.0)	192.3 (96.3, 345.0)	167.5 (53.1, 230.5)	24.8 (17.6, 30.2)	54.8 (49.6, 65.7)	2.3 (1.5, 2.9)	63.2 (49.4, 88.9)	111.0 (79.9, 167.7)	4.8 (3.1, 6.6)*
	D21	62.1 (52.9, 70.0)*^#^	32.9 (29.6, 35.4)*	34.0 (16.0, 55.5)*^#^	55.0 (45.5, 91.0)*	106.0 (57.5, 155.5)	70.0 (32.3, 118.5)	171.4 (60.5, 349.1)	134.9 (35.2, 256.5)	22.7 (17.9, 40.5)	55.0 (44.7, 64.4)	2.0 (1.5, 4.4)	67.4 (48.4, 89.5)	107.0 (80.1, 172.6)	4.2 (3.4, 6.6)*
	*Z*	10.949	11.945	27.332	19.496	1.898	7.423	4.076	5.936	1.440	2.080	2.078	3.774	2.771	14.552
	*p* value	0.012	0.008	< 0.001	< 0.001	0.594	0.060	0.253	0.115	0.696	0.556	0.556	0.287	0.428	0.002

Data are shown as median (interquartile range)

*TP* total protein; *ALT* alanine aminotransferase; *AST* aspartate amino transferase; *ALP* alkaline phosphatase; *γ-GGT* γ-gamma-glutamyl transpeptidase; *TBIL* total bilirubin; *DBIL* direct bilirubin; *PT* prothrombin time; *APTT* activated partial thromboplastin time; *INR* international normalized ratio; *Scr* serum creatinine; *GFR* glomerular filtration rate; *WBC* white blood cells

*Compared with D0, the difference was statistically significant

^#^Compared with D7, the difference was statistically significant

### Treatment effects

The overall efficacy rate was 53.1% among all enrolled 258 participants. Notably, the efficacy of caspofungin was not significantly different between Child–Pugh B and C patients (*P* = 0.208). In addition, no significant differences of efficacy was observed among the 1-week, 2-week, and 3-week treatment groups (*P* = 0.077) (Table 4, Online Resource 1, ESM 2).

**Table 4:** Comparison of efficacy among patients with different Child–Pugh scores

Group	Complete response	Partial response	Stable response	Progression of disease	Death	Efficient (%)
Child–Pugh B (*n* = 67)	31 (46.3)	9 (13.4)	9 (13.4)	10 (14.9)	8 (11.9)	40 (59.7)
Child–Pugh C (*n* = 191)	69 (36.1)	28 (14.7)	22 (11.5)	46 (24.1)	26 (13.6)	97 (50.8)
Total (*n* = 258)	100 (38.8)	37 (14.3)	31 (12.0)	56 (21.7)	34 (13.2)	137 (53.1)
*X*^*2*^	2.150	0.061	0.172	2.448	0.121	1.583
*p* value	0.143	0.805	0.678	0.118	0.728	0.208

Data are shown as frequency (%)

## Discussion

To the best of our knowledge, this is the largest clinical study that examined the safety and efficacy of using standard dose of caspofungin in patients with Child–Pugh B or C cirrhosis. In this study, all 258 patients received a maintenance dose of 50 mg/day regardless of their hepatic functions. Herein, patients in the 1-week group had an increase in serum PT and INR levels at the end of caspofungin therapy, while the WBC level in Child–Pugh C cirrhosis patients from the 3-week group decreased gradually with prolonged caspofungin use but it remained fluctuating within the normal range. The impacts of standard dose of caspofungin on hepatic, renal or coagulation functions in patients with liver cirrhosis other than PT and INR levels were not observed, and no dose interruptions or adjustments due to adverse drug reactions were necessitated over the course of administration.

Caspofungin is predominantly metabolized by liver, with only 1% to 2% of the administered dose being cleared by the kidneys. Therefore, hepatic impairment can potentially affect the plasma concentrations of the drug [11]. The pharmacokinetics of antimicrobial agents in patients with hepatic insufficiency have not been well-studied. In an earlier PK study, a nearly 1.8-fold increase of area under the curve (AUC) of caspofungin was observed in 8 patients with moderate hepatic insufficiency (Child–Pugh score of 7–9) compared with 24 healthy control volunteers after a single intravenous of 70 mg caspofungin. In the multiple-dose study, 8 patients with moderate hepatic insufficiency received a reduced dose of 35 mg/day and 8 healthy control volunteers received the standard dose of 50 mg/day, the experiment results showed that the primary pharmacokinetic parameter, AUCs, was similar in both of these subjects. Based on these results, a dose reduction to 35 mg daily following the 70 mg loading dose was proposed for patients with Child–Pugh B conditions [12]. However, it is important to point out that this study only recruited patients with chronic and stable hepatic insufficiency who did not have an acute episodes of illnesses.

Martial et al. [6] reported a population pharmacokinetic model of caspofungin based on the 21 non-cirrhotic ICU patients with Child–Pugh B, and found that the area under the concentration–time curve over 24 h (AUC_24_) value of a 70 mg loading dose followed by 35 mg in patients with Child–Pugh B was significantly lower (65 mg.h/L) than the typical AUC_24_ value of a standard dose in healthy control volunteers (100 mg.h/L), which would result in a suboptimal drug exposure. Gustot et al. [7] performed a pharmacokinetic study of caspofungin in 20 patients with an acute decompensated Child–Pugh B or C cirrhosis, and confirmed that reducing the maintenance dose from 50 to 35 mg still resulted in undesirably low drug exposure, although the AUC_24_ value, indeed, was slightly elevated in cirrhosis. Several case reports published in recent years also revealed that a significantly higher caspofungin accumulation due to decreased hepatic metabolism was not observed in decompensated Child–Pugh B or C cirrhotic patients [8, 9].

These findings indicated that the impact of liver test abnormalities or evidence of cirrhosis on liver drug metabolism function may be less pronounced than previously thought. Conversely, a recommended dose reduction of caspofungin in acute and unstable cirrhotic patients would probably led to low systemic exposure and treatment failure. A possible explanation for these findings is that decreased levels of plasma albumin may lead to altered pharmacokinetics in cirrhotic patients, as most caspofungin exist in protein-binding formation (97%) [13]. The studies reported by Li et al. [14] and Kurland et al. [15] demonstrated that hypoalbuminemia could increase the fraction of unbound caspofungin in critically ill patients, thereby augmenting its clearance and reducing the AUC. In fact, the mean plasma albumin level we observed in the current study was 30.3 g/L (28.1, 32.9), which was close to the value of 33.70 ± 5.11 g/L reported in the study by Li et al. [14].

Kurland et al. [15] also observed an inverse correlation between higher serum total bilirubin levels and caspofungin clearance, while this finding conversely conflicted with the observations by Li et al. [14]. Similarly, a therapeutic drug monitoring study of cefoperazone/sulbactam, also a highly protein bound drug with cefoperazone primarily excreted through bile and 20–30% renal excretion, conducted in 70 cirrhotic patients has also observed that varied serum total bilirubin levels exerted differential effects on drug clearance. Specifically, lower trough concentration (*C*_min_) of cefoperazone was observed in patients with bilirubin levels at both lower and higher extremes (≤ 26.15 μmol/L and > 99.15 μmol/L), while patients with mid-range bilirubin levels (26.15–99.15 μmol/L) were more likely to achieve the pharmacokinetic/pharmacodynamic target [16]. It might be due to patients with normal bilirubin levels tend to have no or only mild hepatic dysfunction, whereas excessively elevated serum bilirubin may cause a greater proportion of unbound drug concentration through competitive binding to plasma albumin with highly protein bound drugs [17].

The distribution of bilirubin levels likely differed between the study cohorts of Kurland et al. [15] and Li et al. [14], with Child–Pugh B patients accounting for the largest proportion in the former (40/46), and Child–Pugh A patients being the predominant subgroup in the latter (25/42). This disparity may account for the stark contrast in findings between the two studies. In the present study, only 68 (26.4%) patients had bilirubin levels between 26.15 μmol/L and 99.15 μmol/L. In contrast, up to 149 (57.8%) patients had bilirubin levels > 99.15 μmol/L, with 136 classified as Child–Pugh C and 13 as Child–Pugh B. Thus, the clearance of caspofungin may not be lower in Child–Pugh C patients compared to Child–Pugh B patients.

It has been suggested that the dosage of caspofungin should be escalated to achieve adequate exposure. Märtson et al. [18] conducted a prospective study to establish a population pharmacokinetic model for caspofungin in 20 critically ill adult patients with suspected invasive candidiasis and found that the fixed dosing regimens may have contributed to an reduced overall target attainment rate when the body weight were over 80% of median weight, and greater than or equal to 120 kg, and less than or equal to 50 kg. To achieve the AUC target, they suggested using a weight-based dosage regimen with a loading dose of 2 mg/kg on day 1 followed by a maintenance dose of 1.25 mg/kg. Compared to the fixed dosing regimens, this approach may need higher daily doses. Furthermore, Bailly et al. [19] also recommended increasing the loading dose of caspofungin to 140 mg, as this regimen could reach pharmacokinetic–pharmacodynamic targets earlier. In particular, the high-dose caspofungin treatment regimen have demonstrated good tolerability without dose-limiting toxicity, and the maximum tolerated dose of caspofungin seemed to be over 200 mg/day [20, 21].

Available evidence indicates that human albumin (HA) infusion could reduce inflammation and oxidative stress, and optimize haemodynamic status. The latest international position statement recommend that the use of HA in liver cirrhosis patients with infections, especially when combined with large ascites, hepatorenal syndrome and sepsis/septic shock should be considered [22]. A retrospective study conducted by Chen et al. validated through Logistic regression analysis that lower HA levels were associated with higher risks of caspofungin-related hepatotoxicity (OR 1.347; 95% CI 1.166–1.556; *P* < 0.001) [23]. In this study, most patients received HA infusion to maintain plasma albumin levels at 30 g/L or even higher, with the mean dose of 60.0 g (30.0, 110.0) during caspofungin treatment.

Infection activates the inflammatory response and can trigger inflammatory cascades that exacerbates liver damage and the degree of hepatic fibrosis. Anti-inflammatory and liver-protective agents, such as branched-chain amino acid, ursodeoxycholic acid, glycyrrhizin, silymarin, S-adenosyl-L-methionine can alleviate liver injury, promote hepatocyte regeneration, enhance liver detoxification, facilitate bile acid metabolism, and inhibit oxidative stress [24–28]. These treatments should be integrated in the management of hepatic inflammation*.* To maximize inhibiting inflammatory injury, in this study, nearly 99% of patients received anti-inflammatory and liver-protective agents, with 77.9% were treated with a combination of drugs. It's also worth mentioning that we found some patients showed improved hepatic or renal function at the end of caspofungin treatment, such as a decrease in ALT, AST, ALP, γ-GGT, or an increase in TP, albumin which could potentially be attributed to the comprehensive effect of infection control, HA infusion, the treatment of anti-inflammatory and liver-protective.

The efficacy rate in the current study was 53.1%, which was slightly lower than our previous report that examined caspofungin in patients with liver damage (63.0%) [29], but similar to or higher than the average reported efficacy rate in most prior studies (33.0–54.3%) [21, 30–33]. There were no statistically significant difference in treatment efficacy among patients with different Child–Pugh scores and treatment durations (*P* > 0.05).

There are several limitations we must acknowledge. First, the present study had a single-center retrospective design, which restricts the representativeness of the data. However, we sought to maximize the sample size to offset this limitation. Second, therapeutic drug monitoring was not undertaken during caspofungin treatment in the present study, so we cannot conclude whether the observed favorable tolerability was due to adequate drug exposure rather than the large therapeutic window of caspofungin. Third, the number of cirrhotic patients who received the reduced caspofungin doses was too limited to allow us to compare the efficacy between standard and reduced dosing regimens.

## Conclusions

Our findings suggest that standard caspofungin treatment regimen has shown good safety and efficacy without increasing the incidence of drug-related adverse events in Child–Pugh B and C cirrhotic patients. It is necessary to reconsider the optimal dosing in this population, particularly when patients receive intensive anti-inflammatory and liver-protective treatment and aggressive HA infusion. However, we suggest close monitoring of potential side effects of caspofungin, especially coagulation function and WBC levels. Overall, this real-world study conducted in patients with moderate to severe liver dysfunction can serve as complementary evidence to randomized controlled trials in guiding the clinical rational use of caspofungin.

### Supplementary Information

Below is the link to the electronic supplementary material.

Supplementary file1 (DOCX 19 KB)

## Data Availability

The datasets used during the current study are available from the corresponding author upon reasonable request.
