# Supplementary material for: Safety and efficacy of non-reduced use of caspofungin in patients with Child–Pugh B or C cirrhosis: a real-world study
Source: Infection. 2024 Jan 24;52(3):1063–72. doi: 10.1007/s15010-023-02162-0 (PMC11143044; doi:10.1007/s15010-023-02162-0)
Supplement: Supplementary file 1 — Supplementary file1 (DOCX 19 KB) [file 15010_2023_2162_MOESM1_ESM.docx]

**ESM 1 Comparison of patient characteristics between the Child–Pugh B and Child–Pugh C groups**

| Variables | Total  (n=258) | Child–Pugh classification | | *Z/X^2^/T* | *p*-Value |
| --- | --- | --- | --- | --- | --- |
|  |  | B  (n=67) | C  (n=191) |  |  |
| Male, n (%) | 187 (72.5) | 45 (67.2) | 142 (74.3) | 1.282 | 0.257 |
| Age, years, X±S | 57.5 ± 12.0 | 60.5 ± 12.3 | 56.5 ± 11.8 | 2.360 | 0.019 |
| Weight, kg, M [P_25_, P_75_] | 60.0 (51.0, 66.1) | 55.0 (50.0, 62.0) | 60.0 (52.0, 67.0) | -2.533 | 0.011 |
| Period of treatment |  |  |  |  |  |
| 1 week, n (%) | 114 (44.2) | 34 (50.7) | 80 (41.9) | 1.579 | 0.209 |
| 2 weeks, n (%) | 93 (36.0) | 23 (34.3) | 70 (36.6) | 0.116 | 0.734 |
| 3 weeks, n (%) | 51 (19.8) | 10 (14.9) | 41 (21.5) | 1.338 | 0.247 |
| Department |  |  |  |  |  |
| Infectious Diseases, n (%) | 226 (87.6) | 55 (82.1) | 171 (89.5) | 2.527 | 0.112 |
| Gastroenterology, n (%) | 9 (3.5) | 1 (1.5) | 8 (4.2) | 1.071 | 0.301 |
| Hepatobiliary Surgery, n (%) | 5 (1.9) | 1 (1.5) | 4 (2.1) | 0.094 | 0.759 |
| Intensive Care Unit, n (%) | 4 (1.6) | 4 (6.0) | 0 (0) | 11.583 | 0.001 |
| Hematology, n (%) | 4 (1.6) | 3 (4.5) | 1 (0.5) | 5.081 | 0.024 |
| Oncology, n (%) | 3 (1.2) | 1 (1.5) | 2 (1.0) | 0.086 | 0.770 |
| Respiratory, n (%) | 2 (0.8) | 0 (0) | 2 (1.0) | 0.707 | 0.400 |
| Nephrology, n (%) | 2 (0.8) | 1 (1.5) | 1 (0.5) | 0.605 | 0.437 |
| Gastrointestinal Surgery, n (%) | 2 (0.8) | 0 (0) | 2 (1.0) | 0.707 | 0.400 |
| Rheumatology and Immunology, n (%) | 1 (0.4) | 1 (1.5) | 0 (0) | 2.862 | 0.091 |
| Complication |  |  |  |  |  |
| Coronary heart disease, n (%) | 28 (10.9) | 7 (10.4) | 21 (11.0) | 0.015 | 0.901 |
| Hypertension,  n (%) | 46 (17.8) | 19 (28.4) | 27 (14.1) | 6.848 | 0.009 |
| Diabetes, n (%) | 47 (18.2) | 15 (22.4) | 32 (16.8) | 1.057 | 0.304 |
| Haematological malignancy, n (%) | 39 (15.1) | 12 (17.9) | 27 (14.1) | 0.551 | 0.458 |
| Tumour, n (%) | 53 (20.5) | 21 (31.3) | 32 (16.8) | 6.468 | 0.011 |
| Cirrhosis Etiology |  |  |  |  |  |
| Viral, n (%) | 159 (61.6) | 38 (56.7) | 121 (63.4) | 0.923 | 0.337 |
| Alcoholic, n (%) | 26 (10.1) | 5 (7.5) | 21 (11.0) | 0.683 | 0.409 |
| Autoimmune, n (%) | 32 (12.4) | 12 (17.9) | 20 (10.5) | 2.527 | 0.112 |
| Cholestatic, n (%) | 19 (7.4) | 5 (7.5) | 14 (7.3) | 0.001 | 0.971 |
| Other/Unknown, n (%) | 22 (8.5) | 7 (10.4) | 15 (7.9) | 0.428 | 0.513 |
| Diagnostic grades of IFI |  |  |  |  |  |
| Confirmed, n (%) | 114 (44.2) | 37 (55.2) | 77 (40.3) | 4.471 | 0.034 |
| Clinical diagnosis, n (%) | 105 (40.7) | 21 (31.3) | 84 (44.0) | 3.281 | 0.070 |
| Suspected diagnosis, n (%) | 39 (15.1) | 9 (13.4) | 30 (15.7) | 0.200 | 0.655 |
| Site of infection |  |  |  |  |  |
| Intra-abdominal,  n (%) | 99 (38.4) | 21 (31.3) | 78 (40.8) | 1.891 | 0.169 |
| Pulmonary, n (%) | 82 (31.8) | 24 (35.8) | 58 (30.4) | 0.681 | 0.409 |
| Oral cavity, n (%) | 35 (13.6) | 5 (7.5) | 30 (15.7) | 2.875 | 0.090 |
| Digestive tract, n  (%) | 30 (11.6) | 12 (17.9) | 18 (9.4) | 3.476 | 0.062 |
| Blood, n (%) | 5 (1.9) | 3 (4.5) | 2 (1.0) | 3.071 | 0.080 |
| Unknown, n (%) | 7 (2.7) | 2 (3.0) | 5 (2.6) | 0.025 | 0.874 |
| Albumin, g/L, M [P_25_, P_75_] | 30.3 (28.1, 32.9) | 31.9 (29.1, 36.6) | 30.0 (28.0, 32.0) | -3.368 | 0.001 |
| TBIL, umol/L, M [P_25_, P_75_] | 132.4 (46.1, 310.9) | 29.0 (17.5, 63.6) | 208.6 (89.1, 360.4) | -7.956 | <0.001 |
| Anti-inflammatory and liver-protective agents |  |  |  |  |  |
| Unused, n (%) | 3 (1.2) | 3 (4.5) | 0 (0.0) | 8.653 | 0.003 |
| 1 Type, n (%) | 54 (20.9) | 24 (35.8) | 30 (15.7) | 12.126 | <0.001 |
| 2 Types, n (%) | 109 (42.2) | 22 (32.8) | 87 (45.5) | 3.286 | 0.070 |
| 3 Types or more, n (%) | 92 (35.7) | 18 (26.9) | 74 (38.7) | 3.050 | 0.081 |
| Dose of HA infusion during caspofungin treatment, g, M [P_25_, P_75_] | 60.0 (30.0, 110.0) | 40.0 (0.0, 110.0) | 70.0 (30.0, 110.0) | -2.956 | 0.003 |

Data are shown as frequency (%), mean ± standard deviation, or median (interquartile range). IFI invasive fungal infection, TBIL total bilirubin, HA human albumin.

**ESM 2 Comparison of efficacy among different treatment durations**

| Group | Complete response | Partial response | Stable response | Progression of disease | Death | Efficient(%) |
| --- | --- | --- | --- | --- | --- | --- |
| 1 week  (n=114) | 38 (33.3) | 14 (12.3) | 17 (14.9) | 31 (27.2) | 14 (12.3) | 52 (45.6) |
| 2 weeks  (n=93) | 40 (43.0) | 17 (18.3) | 8 (8.6) | 16 (17.2) | 12 (12.9) | 57 (61.3) |
| 3 weeks  (n=51) | 22 (43.1) | 6 (11.8) | 6 (11.8) | 9 (17.6) | 8 (15.7) | 28 (54.9) |
| Total  (n=258) | 100 (38.8) | 37 (14.3) | 31 (12.0) | 56 (21.7) | 34 (13.2) | 137 (53.1) |
| *X^2^* | 2.534 | 1.844 | 1.933 | 3.623 | 0.367 | 5.137 |
| *p*-Value | 0.282 | 0.398 | 0.380 | 0.163 | 0.832 | 0.077 |

Data are shown as frequency (%).
